# Subtle Cognitive Variability in Foetal Alcohol Syndrome Spectrum: Intelligence Profiles and Verbal Fluency Performance Across Diagnostic Categories in Polish Population

**DOI:** 10.3390/jcm15031233

**Published:** 2026-02-04

**Authors:** Przemysław Zakowicz, Teresa Jadczak-Szumiło, Max Brzezicki, Kacper Jędrczak, Zuzanna Wiśniewska, Jarosław Bąbka, Maria Skibińska

**Affiliations:** 1 Department of Neural Engineering and Space Medicine, Collegium Medicum, University of Zielona Góra, 65-001 Zielona Góra, Poland106231@g.elearn.uz.zgora.pl (Z.W.); mariaski@ump.edu.pl (M.S.); 2 Item Psychological Centre in Żywiec, 34-300 Żywiec, Poland; 3 Centre for Child and Adolescent Treatment in Zabór, 66-003 Zabór, Poland; 4 Jesus College, University of Oxford, Oxford OX1 3DW, UK; 5 Department of Paedagogy, University of Zielona Góra, 65-762 Zielona Góra, Poland; 6 Department of Genetics in Psychiatry, Poznan University of Medical Sciences, 60-806 Poznań, Poland

**Keywords:** foetal alcohol syndrome, cognitive functions, special pedagogy

## Abstract

**Background/Objectives:** The foetal alcohol syndrome spectrum is linked with neurodevelopmental delay and cognitive and educational problems. Direct consequences of prenatal alcohol exposure include impaired processes of neural migration and brain development. Among the important features present in affected children are impaired communicational skills and intelligence. **Methods:** Here we presented the case–control comparison of 124 children with foetal alcohol syndrome spectrum disorder (FAS: 62 (50%), pFAS: 34 (27.42%) and ARND: 28 (22.58%)) and 53 healthy controls regarding intelligence quotient and a verbal fluency task. The verbal and non-verbal intelligence was measured using the WISC-R scale, and the verbal fluency task encompassed phonemic, semantic and categorial assessment in 15 and 60 s; we used the authors’ parental/caregiver survey to determine risk factors. In statistical analysis both methods of classical parametric/non-parametric tests and machine learning algorithms were used. **Results:** Foetal alcohol syndrome spectrum patients were clearly distinguished from healthy controls regarding total verbal and non-verbal intelligence, as well as all three categories of verbal fluency (*p* < 0.01). ML methods distinguished an FAS group with 0.49 precision and 80% recall and for pFAS and ARND diagnoses we obtained: 0.50/0.33 precision and 3%/7% recall. None of the parameters analysed in our study differentiated foetal alcohol syndrome, partial foetal alcohol syndrome and alcohol-related neurodevelopmental disorders. **Conclusions:** Children with foetal alcohol syndrome spectrum disorder markedly differ from healthy control subjects in intelligence and verbal fluency. The diagnostic sub-types of foetal alcohol spectrum are not clearly defined in obtained neuropsychological and clinical data.

## 1. Introduction

Prenatal alcohol exposure (PAE) may lead to teratogenic effects including facial dysmorphia and internal organ malformation [1]. The global epidemiological burden of pregnancy alcohol exposure varies between geographical regions and reaches up to 9.8% worldwide [2,3]. The prevalence of foetal alcohol syndrome (FAS) is estimated to be 9.4 per 10,000; regarding the whole spectrum of alcohol-related conditions (FAS spectrum, FASD), epidemiological data suggest even an 8-fold greater epidemiology. The epidemiological burden of FASD in the Polish population may reach 80–100 per 10,000 [4].

Despite the frequency of alcohol-related foetal conditions, the clinical process of diagnosis is not based on the same criteria in different countries. Criteria focus mainly on facial dysmorphology and neurodevelopmental disorder [5].

FASD may be treated as the model neurodevelopmental disorder from many points of view. Typically, neurodevelopmental conditions, like autism spectrum disorder (ASD), attention-deficit hyperactivity disorder (ADHD), or intellectual disability (ID), are perceived as multifactorial brain-affecting disorders with a genetic and environmental component. In the case of FASD, despite the genetic burden, it is always connected with PAE. Additional risk factors for FASD are: duration of alcohol exposition during pregnancy, socio-economic status, binge drinking and psychoactive substance exposure [6]. The known aetiological factor enables the tracking of different trajectories of disorders in brain development. Neuroimaging studies have revealed altered brain morphology in FASD children. The most replicable findings include: corpus callosum thickening, reduced white matter integrity and reduced cerebellar volume [7]. These findings may correspond to altered functional connectivity, fractional anisotropy changes and finally the neuropsychological profile of patients’ developmental deficits.

Toxicity of PAE was linked with a plethora of neuronal processes, like neuronal migration, improper network shaping and white matter growth. The particular impact of PAE was clearly described for neural crest cell migration processes [8]. Consumption of alcohol during the early stages of pregnancy interacts with the neurulation process regarding different molecular pathways. Animal model studies identified potential mechanisms affecting the development of the brain following PAE. Based on preclinical data, PAE results in a broad spectrum of brain damage, depending on the gestational age and the dose of consumed alcohol. Most data from rat models of FASD are from the third trimester, encompassing the prefrontal–hippocampal connection, thalamus and surrounding structures, as well as cerebellum damage. Consumption of alcohol during the third trimester was linked to executive functioning deficits due to fronto-hippocampal damage. In the study by Rosenblum et al. [9], rats exposed to alcohol performed significantly worse at the executive functioning task with aberrant oscillatory activity of the medial prefrontal cortex (mPFC) and hippocampus. Authors suggest that third-trimester PAE may impact neuronal oscillations in crucial structures for executive functioning and memory processing. These changes may correspond to neuronal migration processes and proliferation due to, e.g., induction of chemokine pathways, particularly CCL5 and CXCL16 [10]. The third trimester is also perceived as the developmental window for cerebellum damage [11]. In the rat model, toxicity of PAE was linked with proapoptotic pathways, like the voltage-dependent anion channel (VDAC) and adenine nucleotide translocator (ANT) [12]. Similar observations were made for anterior thalamic neurodegeneration [13,14]. In summary, rodent models suggest aberrant prefrontal–thalamic–hippocampal connectivity following PAE. Such changes may be responsible for aberrant cognitive flexibility in later life.

Recent data on an FASD children’s group proved the functional disconnection of the fronto-limbic system in functional neuroimaging [7]. As it was proven, the aberrant pattern of activation was clearly correlated with continuous performance task results, which means this system may be responsible for attentional and emotional regulation deficits. Executive function deficits among the FASD population was highly correlated with academic achievements of children [15]. Regarding linguistic processing, children with FASD show significant alterations in cortical structure and organisation, affecting phonological processing, language comprehension and the integration of linguistic and social information (left- and right-hemisphere language deficits) [16].

General neurocognitive development of children with FASD presents language and communication disorders in connection with executive functioning problems. Language deficits are also one of the most frequently reported neuropsychological deficits in the broad population [17]. A case–control study revealed significant differences between FASD and healthily developing children regarding receptive and expressive language. As it was stressed by Poth et al. [18], FASD children did not differ in expressive language but showed remarkably deficits in functional and social communication. These data indicate a different developmental pattern of language processing, including meta-language. The concept of meta-language plays a crucial role in higher-order language functions, including the interpretation of metaphors, irony, and sarcasm, as well as the regulation of prosody and emotional tone. It also supports metalinguistic awareness by enabling the speaker to infer communicative intent, monitor discourse coherence, and adapt language use to social contexts [19]. In meta-language processing, the non-dominant brain hemisphere is engaged. Meta-language disorders are highly linked to the theory of mind (ToM) and pragmatic use of communication. Such deficits may lead to social isolation and communicational problems [20].

In this study, we aimed to compare neurotypical (healthy control, HC) children with FASD children regarding the neuropsychological parameters of (i) Wechsler Intelligence Quotient (WISC-R) for verbal, non-verbal and total intelligence and (ii) a verbal fluency task including a phonemic, semantic and conceptual category. We aimed to track differences between the FASD group and healthy controls in cognitive performance as well as find the distinction between FASD sub-categories (foetal alcohol syndrome: FAS, partial foetal alcohol syndrome: pFAS, alcohol-related neurodevelopmental disorder: ARND) regarding IQ profile and verbal fluency.

## 2. Materials and Methods

### 2.1. Participants and Protocol

The study was conducted as the quality improvement data analysis from a broader scientific project, Psychological diagnosis of children with foetal alcohol syndrome (FAS): Possibilities of using the WISC-R scale in psychological diagnosis (Registration No.: N N106 346340), carried out under the supervision of Prof. Dr. Hab. Elżbieta Hornowska. The comparison analysis obtained clinical data of 124 patients diagnosed with the foetal alcohol spectrum disorder and 53 healthy controls aged 7–16 years old. The diagnosis was made with the use of:

(i) Facial dysmorphia assessment (4-Digit code assessment): philtrum, lip red zone and the palpebral fissure length.
(ii) Parent/caregiver questionnaire: 96 items; author survey included: data on pregnancy, childbirth and early developmental milestones, education and behavioural issues, medical data and family burden.
(iii) Psychometric analysis including: verbal fluency task (VFT) and Wechsler Intelligence Scale for Children Revised (WISC-R).

Children were recruited for participation in the study, with parental consent, from among routine patients of the Neurodevelopmental Diagnostic Outpatient Clinic Item in Żywiec, Poland. The recruitment procedure included informed consent of the patient and/or the caregiver due to local law regulations. The study was approved by the ethical committee at Adam Mickiewicz University in Poznań, Poland (Ref. no: 474/2010). The recruitment period was between 2010 and 2012, and collection of the database for analysis and re-evaluation analysis was performed in 2025. The diagnostic protocol involved multi-disciplinary collaboration of child development professionals (developmental psychologist, neuropsychologist, children and adolescent psychiatry specialist, and physiotherapist), and clinical genetic consultation was carried out if needed to exclude potential genetic syndromes. Inclusion criteria to the study group involved diagnosis of FAS, pFAS and ARND. The clinical diagnosis of FASD was structuralized using the National Institute on Alcohol Abuse and Alcoholism (NIAAA) diagnostic framework [21], including:

- Foetal alcohol syndrome (FAS): the most severe form of foetal alcohol spectrum disorder, characterised by growth deficiency, distinctive facial dysmorphology, and central nervous system abnormalities.
- Partial foetal alcohol syndrome (pFAS): diagnosed when some but not all cardinal features of FAS are present, typically including a history of prenatal alcohol exposure, selected facial anomalies, and either growth restrictions or CNS dysfunction.
- Alcohol-related neurodevelopmental disorder (ARND): this refers to neurocognitive and behavioural impairments (e.g., deficits in memory, attention, judgement, and impulse control) linked to prenatal alcohol exposure, without the facial dysmorphology seen in FAS/pFAS.

Children were assigned to the control group from healthy volunteers according to the following criteria: absence of facial dysmorphia; no evidence of brain damage; no difficulties in social functioning, confirmed through interview and observation; and maternal report of no alcohol consumption during pregnancy.

### 2.2. Dysmorphological and Psychological Examination

Facial dysmorphia was assessed using evaluation of the philtrum and upper lip vermilion according to the Likert scale from the CODE 4 manual (Asthley, 2004) [22]. Palpebral fissure length was compared to American percentile norms for the Caucasian population. Measurements of palpebral fissure length were taken with a ruler, and all assessments were performed directly rather than from photographs of the children.

Psychological examination protocol included:

Parental/caregiver survey: Parental or caregiver interviews were conducted using a structured questionnaire designed to obtain information on maternal pregnancy course and child development. The form included 96 items, both closed and open-ended, covering birth data (date, weight, and height), current growth parameters, pregnancy history, and—critically—maternal alcohol consumption during pregnancy.

The WISC-R battery, the most widely used tool for assessing intelligence and cognitive abilities in Poland (Hornowska, 2001) [23], was employed as the primary research instrument, supplemented by qualitative analysis of responses. Although intelligence tests do not differentiate difficulties associated with FAS, pFAS, and ARND, WISC-R was chosen given its routine use in psychological assessment of children with school problems.

The verbal fluency task (VFT) was assessed using three one-minute trials: initial letter fluency (ILF), requiring children to generate words beginning with the letter ‘k’, and two semantic category fluency (SCF) tasks, involving the naming of animals and sharp objects. The procedure evaluates lexical retrieval, semantic memory, and executive functions, particularly attention. Performance was analysed both by total word count and by the number of words produced in the first 15 s, which provides additional insight into automatic versus controlled cognitive processing [24]. Verbal fluency was assessed phonemically (‘objects beginning with the letter K’), semantically (‘animals’), and conceptually (‘sharp objects’) with analysis based on the number of words generated during the first 15 s and across the full 60 s trial.

### 2.3. Statistical Analysis

The Kolmogorov–Smirnoff test was used to check the normality of the data. For the data showing standard distribution, we used a *t*-test, and for non-parametric analysis we used the Mann–Whitney U-test and Kruskal–Wallis ANOVA. The significance level was set at *p* < 0.05. The statistical analyses were performed using Statistica v13 software (StatSoft, Krakow, Poland). Machine learning analyses were conducted using a standardised preprocessing pipeline designed to minimise the influence of confounding variables and isolate neuropsychological signals. All features were standardised (μ = 0, σ = 1) to ensure comparability across measures and to allow logistic regression coefficients to reflect diagnostic relevance rather than raw numerical scale. Model performance was evaluated using Leave-One-Out Cross-Validation (LOOCV), a rigorous validation strategy appropriate for small clinical datasets. Two supervised learning algorithms were implemented: a Random Forest classifier (50 trees, maximum depth = 5) and an L2-regularised multinomial logistic regression (C = 0.1, max_iter = 1000). Feature importance in the Random Forest model was quantified using mean decrease in impurity, reflecting each variable’s contribution to diagnostic partitioning across the ensemble of trees. In logistic regression, importance was defined as the mean absolute coefficient across all class-specific decision boundaries, capturing the aggregate discriminative strength of each feature under regularisation constraints. Analyses were performed in two configurations: (i) a full-sample model assessing discrimination between typically developing individuals and the FASD spectrum and (ii) a restricted model evaluating finer-grained differentiation among FAS, pFAS, and ARND.

## 3. Results

### 3.1. FASD Children and Healthy Control Group

The FASD group comprised 79 males and 45 females, with a mean age of 10.7 years (SD = 2.64). Detailed clinical and demographic data are presented in Table 1. Regarding standard distribution variables (*t*-test), we obtained significant differences between FASD patients and HCs in: the semantic verbal fluency task (*p* < 0.01), total IQ (*p* < 0.01) and verbal IQ (*p* < 0.01).

Analysis of variables without standard distribution (Mann–Whitney U’) obtained significant differences in: Phonemic Verbal Fluency (after 60 s: *p* = 0.01; after 15 s: *p* < 0.01), the semantic verbal fluency task (after 15 s: *p* < 0.01), conceptual verbal fluency Task (after 60 s: *p* < 0.01; after 15 s: *p* < 0.01) and non-verbal IQ (*p* < 0.01).

The parental/caregiver survey showed suitable consistency (Cronbach’s α = 0.89 for total difficulties; α = 0.78 for cognitive-practical sub-scale). We obtained significant differences in both the total and cognitive-practical sub-scales of the questionnaire (*p* < 0.01).

### 3.2. Comparison Between FAS, pFAS and ARND

The diagnostic categories among the whole studied population (n = 124) included: FAS: 62 (50%), pFAS: 34 (27.42%) and ARND: 28 (22.58%).

For parametric analysis, the post hoc Tukey test revealed no significant differences between FAS, pFAS and ARND groups regarding: Phonemic Verbal Fluency (60 s: *p* = 0.34; current F effect = 1.08), semantic verbal fluency (60 s: *p* = 0.55; current F effect = 0.60), total IQ score (*p* = 0.67; current F effect = 2.76), verbal IQ score (*p* = 0.21; current F effect = 1.57) and non-verbal IQ score (*p* = 0.08; current F effect = 2.48).

For non-parametric variables, Kruskal–Wallis ANOVA revealed no statistically significant differences between the studied subpopulation of FASD diagnoses for: Phonemic Verbal Fluency (15 s: *p* = 0.34; H = 2.15), semantic verbal fluency (*p* = 0.34; H = 2.10), and conceptual verbal fluency (60 s: *p* = 0.06, H = 5.63; 15 s: *p* = 0.0.25, H = 2.74).

#### Machine Learning Analysis

Two winning models were chosen: Random Forest and logistic regression to classify between (i) affected patients (FAS, pFAS, and ARND) and healthy controls and (ii) between FAS, pFAS and ARND diagnoses.

**Figure 1:**
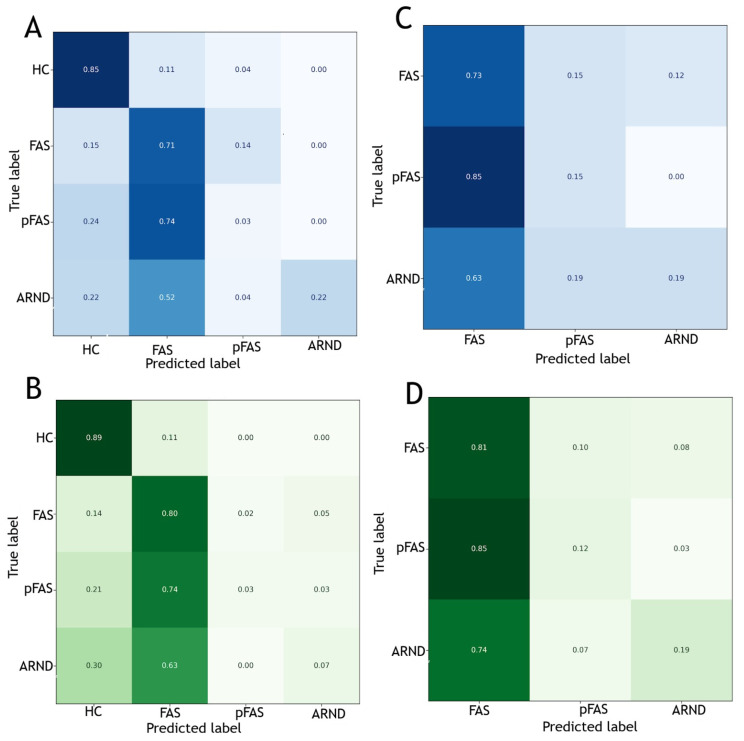
Recall confusion matrix for Random Forest (**A**,**C**) and logistic regression (**B**,**D**). (**A**) Healthy controls (HCs) and FASD (FAS, pFAS, and ARND) classification—Random Forest. (**B**) Healthy controls (HCs) and FASD (FAS, pFAS, and ARND) classification—logistic regression. (**C**) FASD classification (FAS, pFAS, and ARND)—Random Forest. (**D**) FASD classification (FAS, pFAS, and ARND)—logistic regression. The darker the cell colour, the higher the proportion of cases assigned to that field—whether correctly or incorrectly.

(i) In the classification between FASD patients and HCs, for Random Forest we obtained: 0.66 precision and 85% recall for healthy controls and 0.48 precision and 71% recall for FAS. The model showed weak ability to classify pFAS and ARND diagnoses (pFAS: 0.08 precision, 3% recall; ARND: 1.00 precision, 22% recall). For logistic regression we obtained 0.67 precision and 89% recall for HCs and 0.49 precision and 80% recall for FAS; similarly, for pFAS and ARND diagnoses we obtained: 0.50/0.33 precision and 3%/7% recall.
(ii) In the classification between FASD diagnoses, for Random Forest we obtained 0.44 accuracy, with the best recall of 73% for FAS diagnosis, 19% recall for pFAS and 15% for ARND. The most important coefficients were: total IQ, conceptual verbal fluency and semantic verbal fluency. In logistic regression we obtained 0.47 accuracy, with the best recall (81%) for FAS, 12% for pFAS and 19% for ARND. The most important coefficients for the prediction were similarly total IQ and semantic/conceptual verbal fluency. For the detailed data see the figure below (Figure 1).

## 4. Discussion

Here we present complementary analysis of both classical statistical methods and machine learning classification models among FASD patients and a healthy control population. Obtained results showed that clear distinction between populations was obtained only for FASD and HC children with no significant parameters specific to FAS, pFAS and ARND. These results show that widely used clinical tools like intelligence quotient and a verbal fluency task may potentially serve as diagnostic support for FASD diagnosis but are not suitable for FASD sub-types.

In our study, all of the categories of verbal fluency measurement and both time points of assessment (15 s and 60 s) were significantly different between alcohol-exposed children and healthy controls; VFT parameters were also used as crucial coefficients in the ML classification model. A verbal fluency task was used in previous studies as an component of complex neurocognitive batteries but without specific relationships with sub-types of FASD [25,26]. Correct performance of these tasks depends on the ability to initiate searches and retrieve data from one’s vocabulary store or semantic memory system, as well as on effective executive functioning, including attention. Thus, the verbal fluency test is a good assessment tool for individuals with central nervous system damage, including damage to the frontal lobes [24]. The ability to generate words following the instruction requires an complex interplay between linguistic areas of the temporo-frontal cortex and executive functions. The VFT was linked to different neuropsychiatric conditions like neurodegeneration disorders, attention-deficit hyperactivity disorder, developmental dyslexia and language impairment [27]. In this test, not only is the total number of words the child produced within the allotted time counted but also the number of words they ‘generated’ during the first fifteen seconds, because the number of words produced in the first fifteen seconds provides additional insight into cognitive processes [24]. During the first fifteen seconds, children and adults list a ready, easily accessible set of frequently used words that are automatically activated for retrieval. Over time, this set becomes exhausted. Searching for new words requires greater effort. This search phase is usually less efficient and therefore depends more heavily on executive functions [24].

Executive function deficits are generally linked with foetal alcohol syndrome and are decisive factors regarding poor ability to maintain attention and regarding decision-making and social functioning [25]. Overlapping communicational and executive function deficits may be the main source of suffering and social isolation among this group [28]. Our results partially overlap with previous studies [25,26]. Research on an American population showed diminished ability of FASD children to produce words in the phonemic but not semantic part of the task [26]. Similar data were also replicated in previous studies [29]. In our model, the significant alterations were noticed in all kinds of verbal fluency, which may be explained by broader population data, as well as linguistic differences depending on ethnicity. In the version of the VFT we used, the third category of task, ‘conceptual’, may be specific to the Polish population: the word ‘sharp’ in the Polish language has two denominators, one for sharp objects, like a knife, needle and similar, and one for ‘spicy ones’, like chilli or pepper. Hence, there is an additional measure of flexibility in language production enabling the shift between two semantic categories. However, verbal fluency task deficits not specific to the FASD population may be a crucial tool in screening and diagnosis due to easy application in primary care systems with no need for specialist psychiatric professionals. The task also shows a potential source of communicational defects among FASD adolescents, where the direct coincidence of word production is linked with daily life communication abilities [25].

An additional factor distinguishing the FASD population from healthy controls is both verbal and non-verbal intelligence measured by the WISC-R scale. In the study model we decided to use WISC-R instead of other scales, like Stanford–Binet, due to unobvious features specific to the FASD population. WISC-R provides clear distinction between verbal and non-verbal communication and may thus be a better screening tool for FASD children [30,31]. An additional value of the scale is that it can work with the higher heterogeneity of cognitive profiles found among foetal alcohol spectrum children, especially regarding executive deficiencies (planning, inhibition, and flexibility) [32]. WISC-R may also provide value regarding differential diagnostics between FASD and ADHD or ASD [33].

We found no significant values providing a distinction between three sub-types of FASD diagnoses (FAS, pFAS, and ARND). In the studied population, both statistical methods showed no parameters clearly defining each diagnostic category. Differential diagnostics between FAS, pFAS and ARND is linked with the documented alcohol exposure in pregnancy for pFAS and ARND (PAE), dysmorphic features (complete three crucial dysmorphias for FAS, ≥2 for pFAS) and neurobehavioral deficits [34]. Diagnostics categories do not directly stress the value of verbal fluency or intelligence quotient for particular sub-types of FASD, hence the lack of a concrete cognitive profile for pFAS and ARND. The other explanatory route may be the consequence of controversy surrounding FAS sub-types in clinical guidelines, largely due to their inconsistent operational definitions, variable clinical thresholds, and limited neurobiological justification [34,35]. The limited discriminability between pFAS and ARND in both classical statistical analyses and machine learning models suggests that cognitive and verbal fluency measures alone may not sufficiently capture the neurodevelopmental distinctions between these sub-types. Future classification models could benefit from executive function, adaptive, or neurobiological markers, including neuroimaging-derived features, which may provide greater sensitivity to sub-type-specific patterns.

## 5. Conclusions

Novel methods of data analysis with the use of machine learning may provide a more independent approach to foetal alcohol spectrum and minimalize the risk of inter-rater bias [36]. In the analysis we performed, we did not obtain relevant distinguishing features through our clinical review of risk factors in the parent/caregiver survey enabling the stratification of diagnostic sub-categories. The study is not free from limitations regarding (i) non-representative sex and age distribution among the studied population of FASD and HC children, as well as (ii) the unequal sizes of studied populations, both regarding FASD and HCs, and FAS/pFAS/ARND. (iii) Although all neuropsychological scores were age-standardised according to Polish norms, variability in chronological age across groups may still be prone to developmental variance. Future analyses may benefit from incorporating age as a covariate (e.g., ANCOVA or regression-based adjustment) or age-controlled machine learning models. (iv) Future research should examine the cross-cultural profile of verbal fluency deficits in FASD using harmonised multilingual protocols. (v) The cross-sectional design of the present study limits the ability to infer developmental trajectories among the FASD population.

## Figures and Tables

**Table 1:** Clinic-demographic data and FASD/HC comparison.

	FASD	HC	*p*
**Sex (M/F)**	79/45	28/25	0.17 *
Mean age (±SD)	10.7 (±2.64)	12.0 (±2.97)	**0.01** *
VFT—Phonemic 60 s	6.36 (±3.90)	11.13 (±5.48)	**<0.01** *
VFT—Phonemic 15 s	2.53 (±1.73)	4.66 (±2.48)	**<0.01** *
VFT—Semantic 60 s	13.13 (±4.94)	17.74 (±5.30)	**<0.01** **
VFT—Semantic 15 s	5.25 (±2.26)	7.06 (±2.62)	**<0.01** *
VFT—Conceptual 60 s	6.17 (±2.75)	9.04 (±3.67)	**<0.01** *
VFT—Conceptual 15 s	3.16 (±1.43)	4.45 (±1.55)	**<0.01** *
WISC-R Verbal IQ	86.90 (±17.77)	120.70 (±12.33)	**<0.01** **
WISC-R Non-verbal IQ	96.40 (±18.34)	119.23 (±14.11)	**<0.01** *
WISC-R Total IQ	90.44 (±17.95)	118.28 (±12.45)	**<0.01** **

Bolded: significant, * Mann–Whitney U’, ** *t*-test.

## Data Availability

The anonymised dataset supporting the findings of this study is available from the corresponding author upon reasonable request. Although all data have been fully anonymised, they contain sensitive clinical information and therefore cannot be made publicly accessible.

## Abbreviations

The following abbreviations are used in this manuscript:

FASD	Foetal Alcoholic Spectrum Disorder
pFAS	Partial Foetal Alcoholic Spectrum Disorder
ARND	Alcohol-Related Neurodevelopmental Disorder
